# Reactive Oxygen Species Production by Forward and Reverse Electron Fluxes in the Mitochondrial Respiratory Chain

**DOI:** 10.1371/journal.pcbi.1001115

**Published:** 2011-03-31

**Authors:** Vitaly A. Selivanov, Tatyana V. Votyakova, Violetta N. Pivtoraiko, Jennifer Zeak, Tatiana Sukhomlin, Massimo Trucco, Josep Roca, Marta Cascante

**Affiliations:** 1 Departament de Bioquimica i Biologia Molecular, Facultat de Biologia, Universitat de Barcelona, and IBUB, Barcelona, Spain; 2 A. N. Belozersky Institute of Physico-Chemical Biology, Moscow State University, Moscow, Russia; 3 Department of Pediatrics, University of Pittsburgh School of Medicine and Children's Hospital of Pittsburgh, Diabetes Institute, Pittsburgh, Pennsylvania, United States of America; 4 Neuropathology Division, Department of Pathology, University of Alabama at Birmingham, Birmingham, Alabama, United States of America; 5 Institute of Theoretical and Experimental Biophysics, Pushchino, Russia; 6 Hospital Clinic, IDIBAPS, CIBERES, Universitat de Barcelona, Barcelona, Catalunya, Spain; Medical College of Wisconsin, United States of America

## Abstract

Reactive oxygen species (ROS) produced in the mitochondrial respiratory chain (RC) are primary signals that modulate cellular adaptation to environment, and are also destructive factors that damage cells under the conditions of hypoxia/reoxygenation relevant for various systemic diseases or transplantation. The important role of ROS in cell survival requires detailed investigation of mechanism and determinants of ROS production. To perform such an investigation we extended our rule-based model of complex III in order to account for electron transport in the whole RC coupled to proton translocation, transmembrane electrochemical potential generation, TCA cycle reactions, and substrate transport to mitochondria. It fits respiratory electron fluxes measured in rat brain mitochondria fueled by succinate or pyruvate and malate, and the dynamics of NAD^+^ reduction by reverse electron transport from succinate through complex I. The fitting of measured characteristics gave an insight into the mechanism of underlying processes governing the formation of free radicals that can transfer an unpaired electron to oxygen-producing superoxide and thus can initiate the generation of ROS. Our analysis revealed an association of ROS production with levels of specific radicals of individual electron transporters and their combinations in species of complexes I and III. It was found that the phenomenon of bistability, revealed previously as a property of complex III, remains valid for the whole RC. The conditions for switching to a state with a high content of free radicals in complex III were predicted based on theoretical analysis and were confirmed experimentally. These findings provide a new insight into the mechanisms of ROS production in RC.

## Author Summary

Respiration at the level of mitochondria is considered as delivery of electrons and protons from NADH or succinate to oxygen through a set of transporters constituting the respiratory chain (RC). Mitochondrial respiration, dealing with transfer of unpaired electrons, may produce reactive oxygen species (ROS) such as O_2_^−^ and subsequently H_2_O_2_ as side products. ROS are chemically very active and can cause oxidative damage to cellular components. The production of ROS, normally low, can increase under stress to the levels incompatible with cell survival; thus, understanding the ways of ROS production in the RC represents a vital task in research. We used mathematical modeling to analyze experiments with isolated brain mitochondria aimed to study relations between electron transport and ROS production. Elsewhere we reported that mitochondrial complex III can operate in two distinct steady states at the same microenvironmental conditions, producing either low or high levels of ROS. Here, this property of bistability was confirmed for the whole RC. The associations between measured ROS production and computed individual free radical levels in complexes I and III were established. The discovered phenomenon of bistability is important as a basis for new strategies in organ transplantation and therapy.

## Introduction

Reactive oxygen species (ROS) are side products of electron transport in the mitochondrial respiratory chain, the principal component of energy transformation in mitochondria. ROS generation starts with the formation of a superoxide radical (O_2_^−^) as a result of interaction between molecular oxygen and free radicals, e.g. semiquinone (Q^−^): O_2_+Q^−^→O_2_^−^+Q [1]. This extremely active compound can be deactivated in cells, mainly through superoxide dismutase [2]. However, H_2_O_2_ formed in this process can interact with various intracellular compounds to produce ROS. ROS production serves as a metabolic signal [3]–[5]. However, when released in excess under certain stress conditions such as hypoxia/reoxygenation, ROS can also directly damage cells [6]. This destructive function of the electron transport chain represents the main problem in organ transplantation [7] and in many systemic diseases, as diverse as Parkinson disease [8] and diabetes [9]. The problem can be so great that in some organisms disruption of the electron transport chain can be a positive factor in increasing lifetime [10].

Although electron transport and coupled ROS production have been the focus of intensive research, important details are still not understood. There is currently debate regarding the relative contribution of various sites of the respiratory chain to overall ROS production [11], [12] and the factors that may alter this contribution [13]. The use of specific inhibitors can localize the sites of ROS production, but their contribution under normal and stress conditions without inhibitors *in vivo* is not clear. It is generally accepted that electron transport from succinate through complex I to NAD^+^, the phenomenon known as reverse electron transport [14], is important for respiration and ROS production [15], [16]. However, the mechanism of ROS production as a result of electron transfer upstream in the respiratory chain is not understood. Some details of the general mechanism of electron transport, such as the interaction of complex I with quinones that results in translocation of four protons through the membrane and reduction of one ubiquinone molecule per two electrons transported, remain the subject of discussions [17], [18]. Answering these questions will help in understanding the mechanisms of electron transport and coupled ROS production, and will be useful for advances in transplantology and therapy.

The solution to such problems requires not only improvements in experimental techniques and new experiments, but also modification of methods for theoretical analysis. Specifically, kinetic modeling, which is an efficient method for investigating complex systems, still needs to be adopted for the mitochondrial respiratory chain. In fact, kinetic modeling in its classical form has been used for analysis of mitochondrial respiration. However, even the most detailed models [19] could consider only simplified scenarios. Huge number of differential equations is necessary to describe the behavior of respiratory complexes, so an automated procedure is required for their construction. Previously we developed a rule-based methodology for the automated construction of large systems of differential equations for analysis of ^13^C isotope tracing experiments in metabolic flux analysis [20]–[22]. We extended this methodology to the mathematical description of multienzyme complexes, specifically mitochondrial respiratory complex III based on a Q-cycle mechanism [23]. A detailed description of complex III operation revealed that in a certain range of parameters complex III has the property of bistability, where two different steady states exist for the same parameters and the system can reach one or the other, depending on its initial state. Perturbations, such as fluctuations in succinate concentrations or temporal hypoxia, can switch the system from low to high ROS producing steady state. Such a switch explains the damaging increase in ROS production on reoxygenation after hypoxia.

The prediction of bistability for the mitochondrial respiratory chain was based on analysis of the Q-cycle mechanism for complex III. The contribution of other parts of the respiratory chain and linked processes that provide substrates must affect the properties of the respiratory chain. To study mitochondrial respiration as a whole, we extended the model of complex III [23]. The extended model includes the following elements: a detailed mathematical description of complex I; the stoichiometry of electron transport and proton translocation by the respiratory chain; the transmembrane potential; proton leak; oxidative phosphorylation; the TCA cycle that produces NADH and succinate as substrates for complexes I and III; and the transport of TCA cycle metabolites. The objective of this extension to the whole respiratory chain and linked processes was to create a tool for analysis of the basic behavior of the respiratory chain, in particular under conditions defining different fluxes in the forward and reverse directions. The ultimate aim was to reveal characteristics that have not been measured, such as the content of various free radicals, and thus to provide an insight into the relationship between states of the respiratory chain operation and ROS production.

## Results

**Figure 1:**
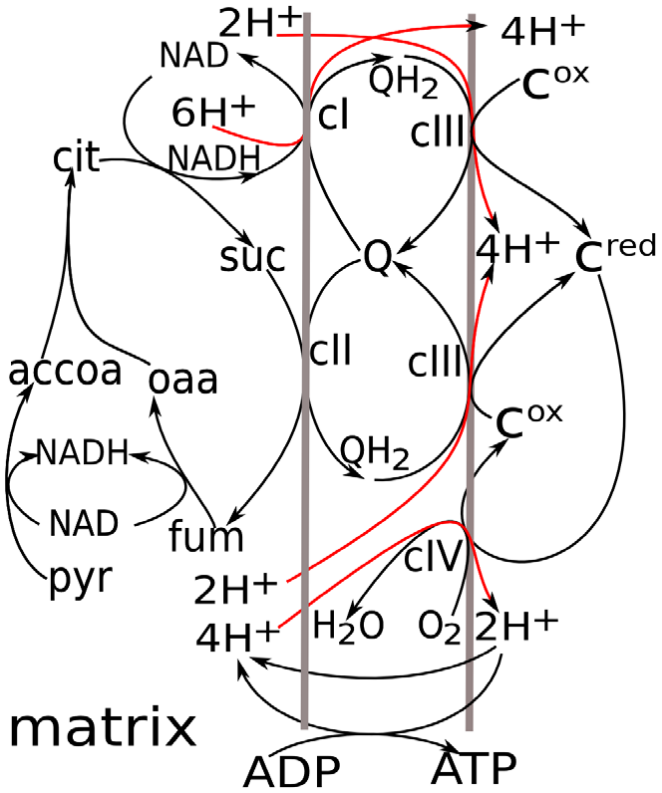
Scheme for mitochondrial respiration and linked processes simulated in the model. Two reactions lead from pyruvate to succinate and further transformation to oxaloacetate reduce NAD^+^ to NADH. The latter is used by complex I to generate a transmembrane electrochemical proton potential (Δμ_H+_) and reduce ubiquinone (Q) to ubiquinol (QH_2_), oxidation of which by complex III also contributes to Δμ_H+_. Complex III reduces cytochrome *c*, oxidation of which by complex IV and reduction of molecular oxygen to H_2_O is also coupled to Δμ_H+_ generation. Oxidation of succinate to fumarate by complex II is coupled to the reduction of ubiquinone and thus fuels complex III. The product of electron transport, Δμ_H+_, is consumed for ATP synthesis.

Figure 1 shows the components of the respiratory chain connected in the extended model. Respiratory complexes I to III are components of the electron transport chain connected through ubiquinone. Complex III is linked to complex IV through reduction/oxidation of cytochrome c. NADH, which is a substrate for complex I, is produced in the TCA cycle. Since the total concentration of NAD^+^ and NADH is conserved, NADH consumption, which fuels electron transport in the respiratory chain, defines the levels of NAD^+^, which is a substrate for several reactions in the TCA cycle. In this way, the extended model links electron transport with central energy metabolism, in particular with the reactions of the TCA cycle.

### Determination of parameters by fitting experimental data

As described in the Methods section, the model of the respiratory chain and linked substrate transport and TCA cycle reactions contains 51 parameters. Out of 22 parameters of complex III, six ratios for forward and reverse rate constants were expressed through midpoint potentials. The order of magnitude for the rate constants for forward electron transport reactions in complex III can be estimated based on previous studies [19]. A qualitative reproduction of measured triphasic dynamics of cytochrome *b*_H_ reduction by succinate in isolated cytochrome *bc*1 complex [24], as described in Text S1 and Figures S1, S2, S3 and S4, provides some restrictions for rate constants for binding/dissociation of complex III with ubiquinone species.

The rates of respiration in the presence of ADP (state 3) or an uncoupler characterize the maximal capacity of the respiratory chain. In the absence of ADP (state 4), the respiration rate is characterized by proton leaks, which must be compensated by respiration. According to our measurements, the respiration rate is 480±40 and 170±30 ng atom O/min/mg protein in the uncoupled and in state 4 in succinate-fueled mitochondria, and 410±30 and 80±20 ng atom O/min/mg protein in mitochondria fueled by pyruvate and malate, respectively.

**Figure 2:**
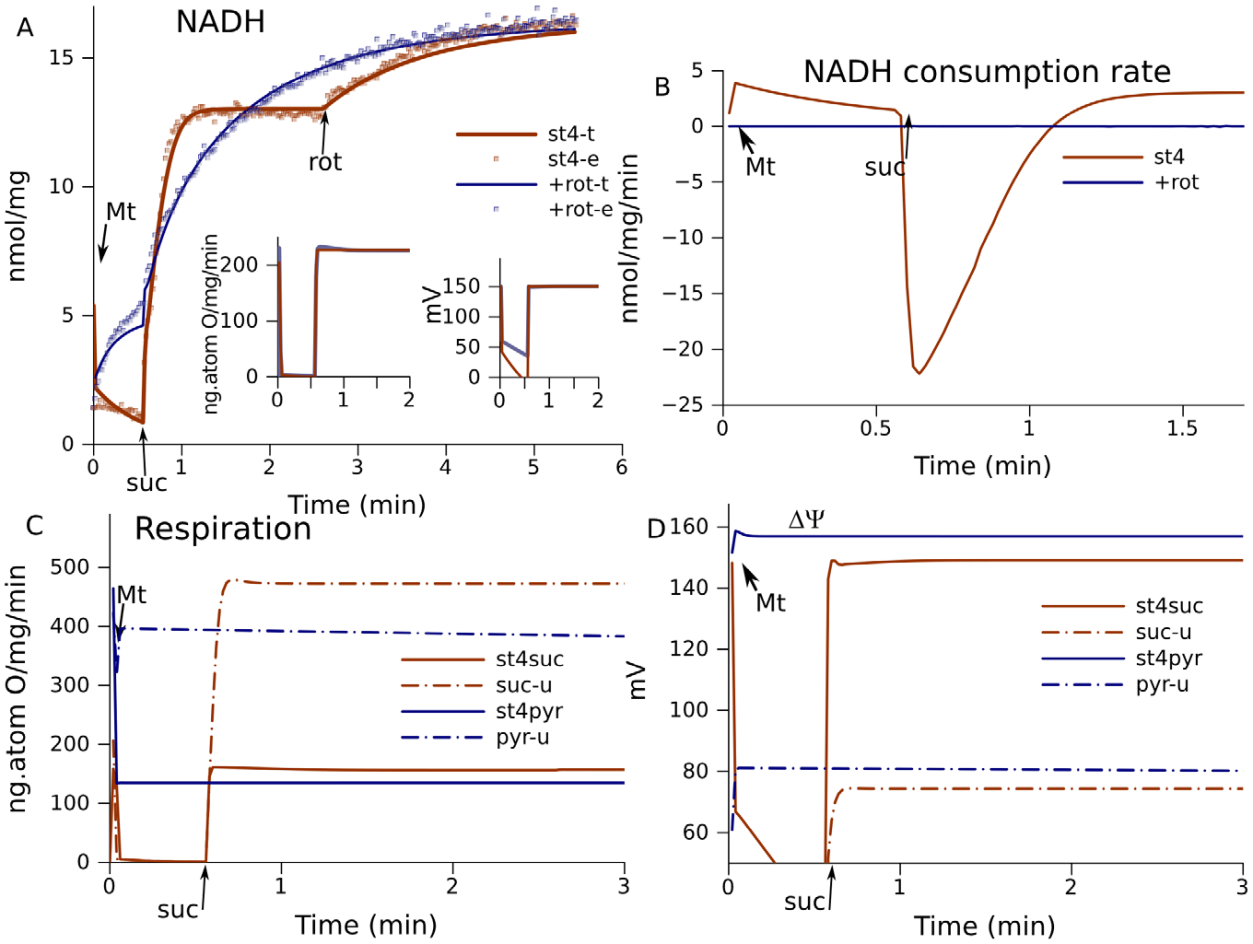
Simulation of forward and reverse electron flows in the respiratory chain. (A) NADH reduction in state 4 in the absence (st4) and presence of rotenone (+rot). Points are experimental data (−e) measured in brain mitochondria fueled by 1 mM succinate, lines are calculated (−t). (B) Reversible electron flow through complex I computed in the simulations shown in (A). (C) Respiration (net forward flux to oxygen) and (D) ΔΨ under different substrate conditions (suc, 1 mM succinate; or pyr, 1 mM malate and 1.5 mM pyruvate ). State 4 respiration (st4) was simulated using the parameters described in Methods. The action of rotenone was simulated by setting *k*_fI5_ = *k*_rI5_ = 0 (rate constants for N2–ubiquinone interactions). The maximal respiration rate (−u, uncoupled) was simulated by setting a high proton leak (*k*_lk_ = 50000 s^−1^). Arrows denote the time of additions of mitochondria (Mt, usually at time 0), succinate (suc), and rotenone (rot) if they ate not present in the medium before mitochondria.

If mitochondria fueled by succinate do not expend the energy of the transmembrane electrochemical potential on ATP synthesis (state 4), succinate oxidation results in fast reduction of intramitochondrial NAD^+^. In the presence of rotenone, an inhibitor of electron transport in complex I, NAD^+^ reduction is characterized by NAD^+^-dependent reactions of the TCA cycle and in particular the forward respiratory flux resulting from succinate oxidation. In the absence of rotenone, reverse electron transport [14] also participates in NAD^+^ reduction, which makes the process much faster (Figure 2A). These data define the rate constants for reverse electron transport.

While succinate fuels complex III through succinate dehydrogenase, the oxidation of malate and pyruvate in the TCA cycle fuels complex I by reducing NAD^+^ to NADH. Respiration under such conditions defines the characteristics of complex I.

To evaluate the model parameters, we used a procedure that simulates all the different types of data listed above for the same set of parameters. The ratio of forward and reverse constants defined by a known midpoint potential or dissociation constant was kept fixed, and the conditions of substrate supply or membrane permeability for protons were changed in accordance with experimental conditions. The procedure fitted all the data by changing the free parameters within the order of magnitude indicated in [19], summarizing and minimizing the deviations in several calculations that simulated measurements. Minimization was performed using a standard stochastic procedure in the global space of parameters as described in Methods.

The best fit reproduces well the dynamics of NAD^+^ reduction measured in brain mitochondria in the presence and absence of rotenone using the same set of parameters (Figure 2A). The insets in Figure 2A show respiration rates and ΔΨ in the presence and absence of rotenone. These characteristics remain practically the same in both conditions. Without rotenone inhibition reversible electron flow through complex I, which fits the experimental data shown in Figure 2A, is directed to NAD^+^ reduction (is negative) only during a short period of time (Figure 2B), although ROS are constantly produced for a much longer time under such conditions [15], [25]. Reverse electron flow is believed to induce excessive ROS production, but evidently these two processes are not correlated.

Rotenone essentially changes the dynamics of NADH measured before succinate addition. It is slightly oxidized by the RC in the absence of rotenone, but slowly reduced in its presence. This reduction is a result of oxidation of internal substrates while electron flow through the RC is blocked. We found that the metabolites of TCA cycle cannot be substrates that provide NADH reduction, because oxidation of TCA cycle metabolites results in much faster initial reduction of NADH. If the parameters of TCA reactions are changed to slow down and reproduce the initial dynamics of NADH, maximal respiration rate with pyruvate becomes inconsistent with experimental data (not shown). Rather, slow oxidation of other metabolites, probably aminoacids or lipids, contributes to NADH reduction. The simulation of such slow oxidation did not prevent NADH oxidation in the absence of rotenone, and reproduced NADH reduction in its presence.

While, in the absence of rotenone, succinate induced much faster NADH reduction due to reverse electron transport, the steady state levels are lower than in the presence of rotenone. The steady state levels are defined by NADH production and consumption in respiration. Rotenone blocks the consumption, therefore NADH levels further increase when rotenone is added after succinate. The model parameters were adjusted without considering subsequent NADH increase and the reproduction of this phenomenon validates the model.

The model reproduces measured maximal and state 4 respiratory electron flows for succinate-fueled mitochondria, as well as for mitochondria fueled by pyruvate/malate (Figure 2C). The change in ΔΨ in the same simulations qualitatively corresponds to known changes measured under such conditions (Figure 2D). The parameters for simulations shown in Figure 2 are listed in Table 1 (column indicated as best fit).

**Table 1:** The 99% confidence intervals of parameters and levels of free radicals.

	max	min	bestfit
k_qp_FS_	267000	117000	200000
k_FS_c1_	1585000	305000	527000
k_qp_bl_	121000	25000	37000
k_bl_bh_	114000	17000	27000
K_bh-qn1_	214000	32000	47000
k_bh_qn2_	1118000	225000	254000
k_qHbnd_	4000	1700	2800
k_qnbnd_	23000	5000	7200
k_qpdis_	9500	1700	2300
k_qhnds_	9500	3300	4100
k_c1c_	290	240	260
k_fI0_	724000	460000	640000
k_fI1_	525000	138000	140000
k_fI2_	816000	255000	770000
k_fI3_	34500	15000	23000
k_fI6_	360000	138000	164000
k_fI8_	721000	143000	205000
k_fI7_	340000	148000	187000
k_tca_	1600	650	710
k_MDH_	1100	270	460
k_spe_	340	140	270
k_me_	0.000382	0.000064	0.000280
k_pyrIn_	1200	500	600
k_sfe_	8.5	2.11	6.48
k_cs_	1300	500	1290
**SQ@Qo**			
st4-suc	0.23318	0.07607	0.16491
+rot	0.23310	0.07602	0.16482
st4-pyr	0.01808	0.01615	0.01756
**SQ@Qn**			
st4-suc	0.12456	0.11350	0.06705
+rot	0.07607	0.06562	0.07120
st4-pyr	0.01293	0.00556	0.00835
**FMNH**			
st4-suc	0.00486	0.00407	0.02525
+rot	0.04094	0.02176	0.03152
st4-pyr	0.00702	0.00284	0.00439
**N2**			
st4-suc	0.135	0.12507	0.07098
+rot	0.07407	0.06320	0.06914
st4-pyr	0.00723	0.00327	0.00482

These intervals were calculated for each parameter separately among the sets of parameters that give χ^2^ below than a fixed threshold [31]. The sets of parameters were found using a stochastic optimization algorithm (Simulated annealing) that minimized the deviation from measured dynamics of NAD+ reduction in the presence and absence of rotenone, and uncoupled and state 4 respiration rates. Only forward rate constants are shown assuming that the reverse constants change proportionally keeping constant the ratio in accordance with respective ΔE_m_ or dissociation constant. The units for rate constants of monomolecular electron transport are s^−1^. For the reactions where protons are bound the concentration of protons for pH = 7 is included in the constant (k′ = k·[H^+^]^2^). These values have the same units s^−1^. Dissociation rate constants are also expressed in the same units. Since concentrations are expressed in nmol/mg prot, rate constants for the bindings are expressed in s^−1^·(nmol/mg prot)^−1^.

These simulations of measured data provide an insight into important hidden characteristics, such as the capacity of ROS production. ROS are produced by the respiratory chain as a consequence of one-electron transfer directly to oxygen from free radicals of electron transporters such as the semiquinone radical (SQ) at the Qo site in complex III [26]–[28] or FMNH [29], SQ bound to complex I [30], or N2 centers [30] in complex I. Simulating the experimental data as presented in Figure 2 the model at the same time simulates the dynamics of these free radicals.

### Qualitative analysis of associations between the overall ROS production and individual radicals

The model describes various states of respiratory complexes formed in the process of electron transport, including those containing free radicals. Such radicals could be responsible for passing unpaired electrons to oxygen thus forming superoxide radicals and other forms of ROS. The contributions of various radicals to ROS production remain unknown; to clarify it we compared measured ROS production and the levels of various free radicals predicted by the model for the same conditions. A similar change in radical content and measured ROS production indicates qualitative accordance between the model and the described process and thus validates the model.

**Figure 3:**
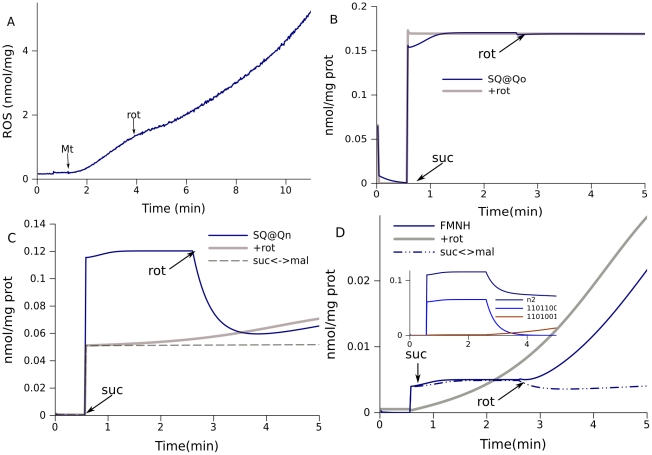
Effect of rotenone on ROS production in mitochondria fueled by 0.5 mM succinate. (A) ROS production measured. (B–D) Model prediction of the content of various free radicals. The dynamics of (B) SQ bound to Qo sites of complex III, (C) SQ at Qn sites of complex I and (D) FMNH were taken from the same simulations of state 4 respiration in succinate-fueled mitochondria for rotenone addition (+rot), either initially or in the course of measurements, as in Figure 2. The simulation marked suc<->mal had a tenfold increased rate constant for this exchange. The inset in (D) shows the dynamics of N2 radicals and species 1101100 and 1101001 for rotenone addition (the digit positions correspond to Qp-Qp-Qn-Qn-N2-FMN-FMN; 1 denotes reduced and 0 oxidized). Arrows indicate the time of additions of mitochondria (Mt), succinate (suc) and rotenone (rot).

It is generally known that inhibition of reverse electron transport by rotenone decreases ROS production in succinate-fueled brain mitochondria [15], . In our measurements, ROS accumulation was inhibited immediately after rotenone addition (Figure 3A). The model predicts that the SQ content at site Qo in complex III in succinate-fueled mitochondria is practically unchanged by the presence of rotenone (Figure 3B) and this remains valid for simulations with any set of parameters describing the data well. This is the reason for the coincidence of intervals for SQ at Qo for the first two types of simulation shown in Table 1. Thus, the ROS-stimulating role of reverse electron transport and the ROS-inhibitory effect of rotenone cannot be explained at the level of complex III. Apparently, reverse electron flow mainly affects complex I by increasing the concentrations of free radicals able to pass electrons to oxygen.

The model predicts that rotenone essentially decreases initial levels of SQ bound on site Qn (Figure 3C), FMNH (Figure 3D), and the content of reduced N2 centers (inset). After an initial decrease, levels of SQ and FMNH increase, in agreement with the acceleration of ROS production measured after initial inhibition induced by rotenone (Figure 3A). The reason for accumulation of free radicals and acceleration of ROS production is the production of malate from succinate, which then reduces NAD^+^ in malate dehydrogenase reactions. This supply of substrate for complex I increases ROS production in rotenone inhibited mitochondria. The increase in the rate constant for malate–succinate exchange eliminates a slow increase in free radical content when rotenone is present (dashed curve in Figure 3C and D). It should be noted that the acceleration of ROS accumulation is not always observed experimentally and this agrees with the predicted disappearance of this slow component after acceleration of malate-succinate exchange. Such similarity of experimental and simulated behavior supports the mechanism accepted for its simulation and in this way validates the model.

The fact that the species of complex I can contain more than one radical makes it more difficult to understand the contribution of each site. In particular, the species 1101001 (the positions of digits correspond to Qp-Qp-Qn-Qn-N2-FMN-FMN), which contain SQ and FMNH radicals, slowly accumulate after inhibition by rotenone. This accumulation defines the dynamics of SQ and FMNH, whereas 1101100 defines the fast component in levels of Qn-bound SQ and reduced N2 (inset in Figure 3D). It is possible that only one of coupled radicals makes the major contribution to ROS production, but in this case the levels of other radicals would also correlate with ROS production. On the other hand, radicals situated inside the same species could interact, so that the specie as a whole produce superoxide. In the considered example the behavior of the whole ensemble of radicals in complex I agrees with the observed effect of rotenone, and this validates the model.

Overall, according to the model predictions, rotenone hardly affects SQ levels in complex III, but initially it significantly decreases the levels of free radicals produced in complex I; this is the reason for the decrease in ROS production induced by rotenone in succinate-fueled mitochondria. The model also explains the subsequent increase in ROS production as a result of the formation of malate in rotenone-inhibited mitochondria.

**Figure 4:**
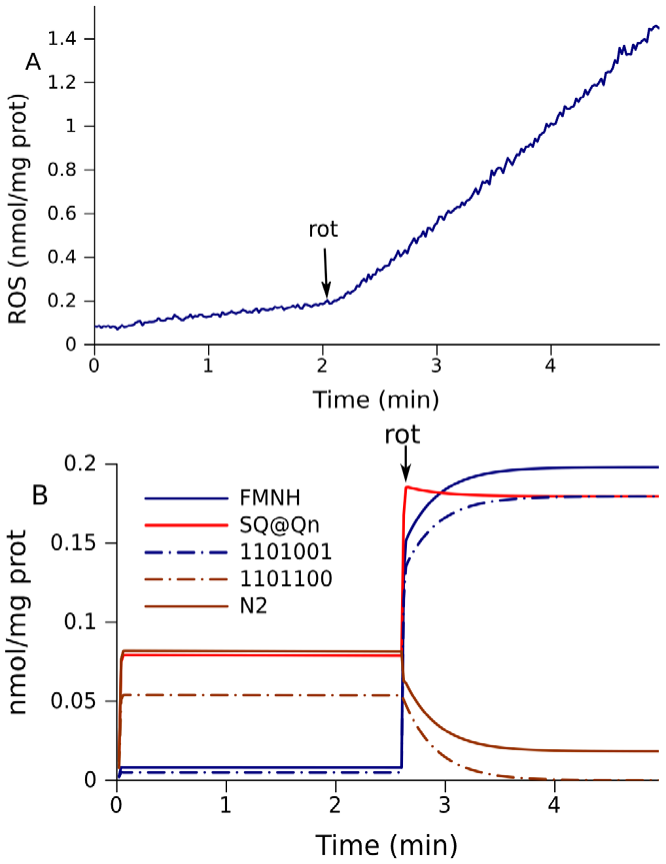
Effect of rotenone on ROS production in mitochondria fueled by 5 mM pyruvate and 5 mM malate. (A) ROS production measured in state 4 respiration and the change on addition of rotenone. (B) Model prediction of free radical levels in a simulation of the conditions for (A). The model parameters are the same as for the simulation shown in Figure 2.

Rotenone induces a large increase in ROS production in pyruvate/malate-fueled mitochondria (Figure 4A). The corresponding simulations show that rotenone greatly increases the levels of FMNH and SQ at Qn site, but decreases the levels of reduced N2 (Figure 4B). Since the changes in N2 disagree with measured ROS production, probably N2 center does not make essential contribution in ROS production under the considered conditions. The same species (1101001) that defined the slow component in the increase in free radicals now change faster and defines the main part of the response to rotenone. Species 1101100 also constitute an essential part of the total radical content, but their levels decrease in response to rotenone in accordance with the decrease of total N2 radical levels.

**Figure 5:**
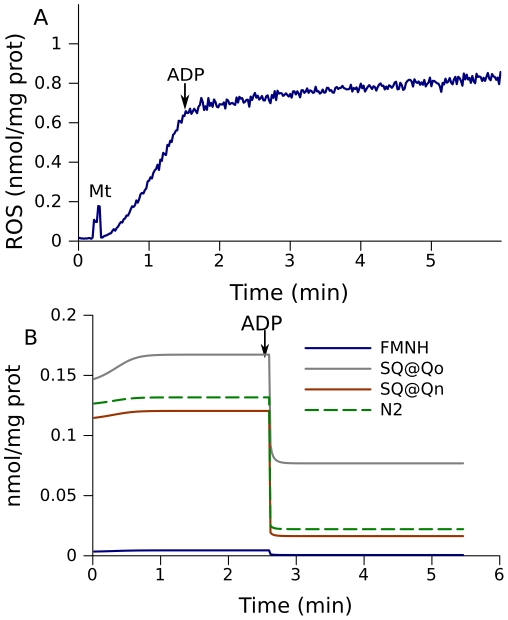
Effect of acceleration of electron transport on ROS production inmitochondria fueled by 3 mM succinate. (A) ROS production measured in state 4 and the change on addition of 1 mM ADP. (B) Model prediction of free radical content. The dynamics of free radical levels in a simulation of the conditions for (A). The model parameters are the same as for the simulation shown in Figure 2.

Stimulation of electron transport by addition of ADP or an uncoupler such as FCCP to succinate-fueled mitochondria results in a decrease in ROS production (Figure 5A). This generally known phenomenon [15] validates the model prediction that the levels of all free radicals decrease when electron transport is stimulated by addition of ADP or an uncoupler.

**Figure 6:**
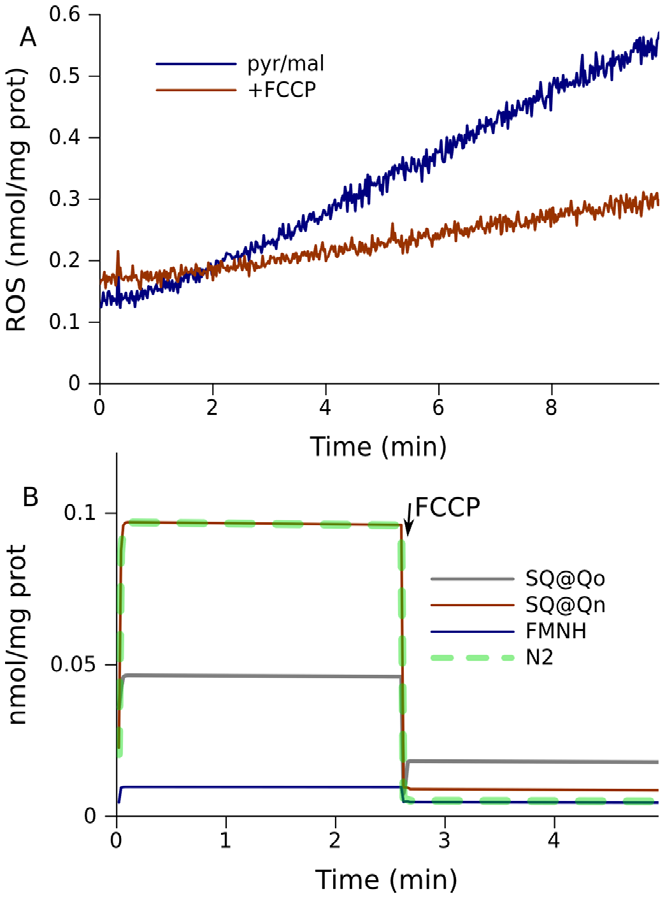
Effect of stimulation of electron transport on ROS production in mitochondria fueled by 5 mM pyruvate and 5 mM malate. (A) ROS production measured in state 4 and in the presence of uncoupler FCCP. (B) Model prediction of free radical content on addition of FCCP. The model parameters are the same as for the simulation shown in Figure 2.

Mitochondria fueled by pyruvate/malate also produce less ROS when electron transport is stimulated by an uncoupler (Figure 6A). Such measurements also validate the model, which predicts a decrease in the levels of free radicals (Figure 6B).

**Figure 7:**
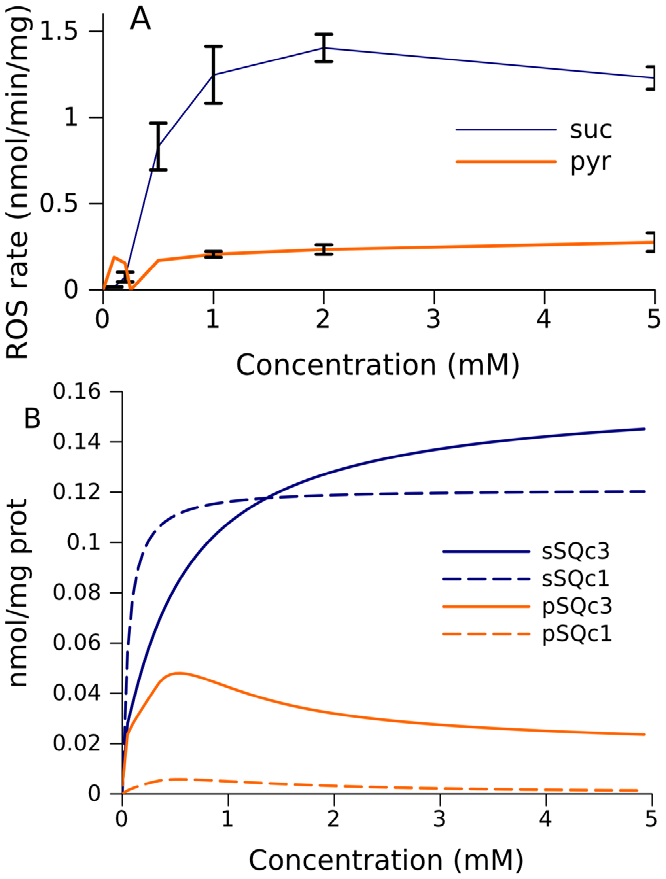
Substrate dependence of ROS production and levels of free radicals. (A) ROS production measured in brain mitochondria. (B) Predicted levels of free radicals in complexes I and III. Succinate (blue curves) or pyruvate (orange) was used as substrate. Semiquinone levels in complexes I (c1) and III (c3) are presented as indicators of ROS production.

At high succinate concentrations, brain mitochondria produce much more ROS than those fueled by pyruvate (Figure 7A). The model also predicts higher levels of free radicals in complex III, as well as in complex I, for mitochondria fueled by succinate (Figure 7B).

Thus, the study of associations between measured ROS production and predicted radical levels in RC revealed qualitative consistency of measurements with all types of radicals and therefore validated the model, or showed a way of discrimination between possible sites of ROS production, and even between possible ROS producing species. However, in the latter case, a special, quantitative study is needed, which currently is beyond of the scope of presented study.

### Prediction of bistability for the whole respiratory chain

**Figure 8:**
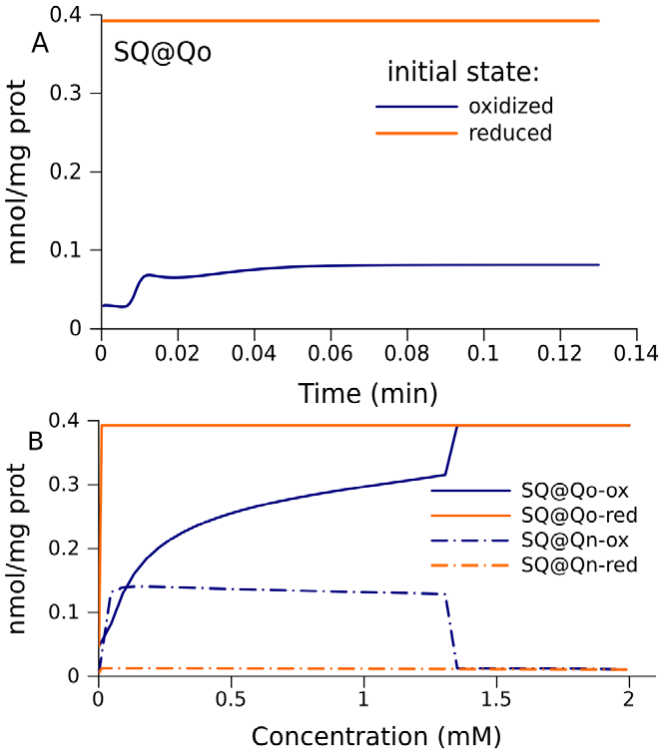
Bistable behavior of the respiratory chain. (A) Predicted dynamics of semiquinones bound to the Qo site of complex III when the system is initially in an oxidized or a reduced state. Substrate concentrations: succinate 0.1 mM, pyruvate 0.25 mM. (B) Steady-state levels of semiquinones bound at the Qo site of complex III and the Qn site of complex I as a function of succinate concentration. The pyruvate concentration for the blue curve is 0.1 mM.

It has been predicted that the Q-cycle mechanism of complex III can in principle induce bistable behavior [23]. The whole respiratory chain considered here, with the parameters that fit the experimental data, also has two different steady states for the same parameters. Figure 8A shows that the SQ content at site Qo in complex III could persist at different values, depending on whether the respiratory chain is initially reduced or oxidized. Figure 8B shows how the steady states for free radicals of complexes I and III change with the external succinate concentration for a set of parameters that reproduces the experimental data described above.

With increase in succinate concentration at some point the system switches to the state with the highest levels of semiquinone radicals at Qo site of complex III. The difference here from the similar curve in Figure 7 is that pyruvate is present in addition to succinate. Once the system is switched to the state of highest SQ content at Qo, it remains in this state even if the succinate concentration decreases back to low values. Thus, if the system is initially in an oxidized state, the steady state SQ levels at Qo depend on the succinate concentration, in accordance with the blue curve in Figure 8B. If the system is initially in a reduced state, it remains in this state until succinate concentrations decrease to the micromolar range. Since complex III is directly connected to complex I through a common substrate (ubiquinone), the bistable behavior of complex III induces bistability in complex I. However, when complex III enters the state with high SQ levels at Qo, SQ levels at Qn decrease (Figure 8B), as well as the levels of other free radicals in complex I (not shown). In some range of succinate concentrations total amount of radicals in the two presented steady states can be similar, but this does not necessary means similar ROS production in the two states since the probability of ROS production can be different for various radicals.

**Figure 9:**
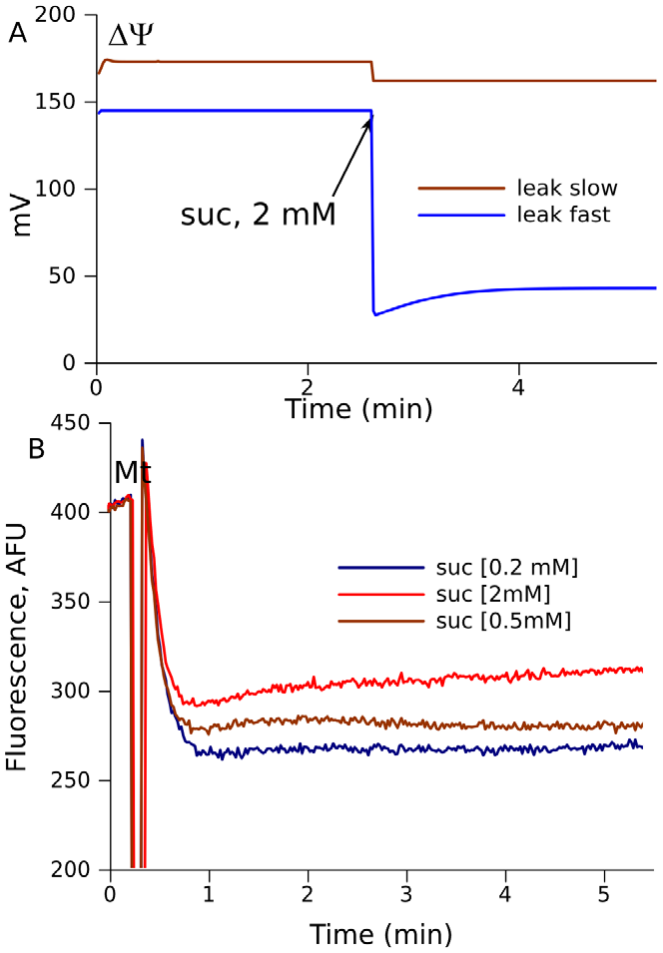
Decrease in ΔΨ on transition from the oxidized to the reduced state. (A) Predicted dynamics of the transition induced by addition of 2 mM succinate to mitochondria initially in the oxidized state in the presence of 5 mM pyruvate, under the conditions of various proton leaks through the inner menbrane, slow leak, k_lk_ = 17000, and fast leak, k_lk_ = 80000 mL/(s·mg prot) (eq.H.1). (B) ΔΨ measured as safranine O fluorescence (lower values correspond to higher ΔΨ) at various succinate concentrations (as indicated). The initial levels of fluorescence (∼400 AFU) is slightly increased in the moment of addition of mitochondria and, then, energization and uptake of the dye results in decrease of fluorescence. The initial levels after the addition of mitochondria correspond to deenergized mitochondria and final corresponds to maximally energized mitochondria.

Thus, bistable behavior remains valid for the extended model of the RC with proton translocation and transmembrane potential (ΔΨ) generation, and with parameters defined by fitting the experimental data and validated by qualitatively similar predicted and measured ROS production. The model predicts also that a pulse of succinate is associated with decrease of ΔΨ. Such counterintuitive decrease of ΔΨ induced by increase of substrate for respiration is shown in Figure 9A. The value of ΔΨ decrease, induced by the same pulse of succinate, can be different, depending, for instance, on membrane leak, as illustrated by two curves in Figure 9A. Measurements of ΔΨ using safranine O fluorescence revealed that the mean ΔΨ at low succinate (0.2 mM) is greater than at high succinate (2 mM) (Figure 9B), thus validating the paradoxical prediction of the model.[Fig pcbi-1001115-g010]

The succinate threshold for a switch to the reduced state depends on the parameters of pyruvate transport and TCA reactions; here we do not investigate the quantitative details with respect to bistability, but emphasize only the qualitative similarity of predicted and measured behavior. With regards to the considered in the previous sections normal “working” steady state, the predicted levels of free radicals are robust with respect to the model parameters, as the next section shows.

### Sensitivity to parameters and robustness of the model predictions

The sensitivity of simulations to variations in model parameters is shown in Table S1 for each type of experimental data presented in Figure 2 (dynamics of NAD^+^ reduction, maximal and state 4 respiratory fluxes). The sensitivity is also listed for simulated levels of free radicals shown in Figure 3. The results indicate that significant changes in some parameters hardly affect the simulations (e.g. *k*_qp_FS_). Evidently, the data do not restrict the parameter values and they could not be defined unambiguously. However, changes in these parameters within the range, for which fitting remains good, do not affect the predictions in terms of free radical levels. The parameters shown in red highly affect the simulations. However, it is possible that different combinations of such parameters could fit the measured data equally well because of mutual compensatory changes. In this case, despite the high sensitivity, the parameters can have a wide range of values for which a good fit is obtained. Confidence intervals rather than sensitivity are used to characterize the robustness of parameter determination.

Different sets in the global space of parameters that fit the experimental data could be identified using our stochastic algorithm for minimization of the objective function χ^2^ (sum of squares of deviations from measured data normalized by standard deviations). The algorithm identified confidence intervals for parameters based on fixed thresholds of χ^2^ [31].

Table 1 shows the 99% confidence intervals for the free parameters. The ranges for which the values give a good fit to the data are large. Thus, even though the measurements cover various modes of respiratory chain operation, the data do not restrict the parameters sufficiently to define them unambiguously. Various sets over a wide range of parameters can describe the data equally well. However, the situation is different for free radical levels predicted for the simulated experimental conditions. Table 1 lists intervals for predicted free radical levels simulated using the parameters sets that fit the data with χ^2^ that is below the threshold. The confidence intervals for free radical levels are generally much narrower, so the predicted values are more robust. Although the intervals for SQ at Qo sites in succinate-fueled mitochondria are relatively large, they are clearly almost the same for both conditions (with or without rotenone). This result agrees with data indicating that the SQ content at Qo practically shows no dependence on the presence of rotenone (Figure 3B). The levels of all free radicals in complex I under the conditions for the first two simulations are very robust, despite the high parameter variability. If the parameters give a good fit, the model predicts similar levels of complex I radicals. Although the intervals are relatively large under the third condition (pyruvate/malate supply), it is evident that they are much lower than the intervals for the condition of succinate supply, as well as the levels of radicals in complex III.

## Discussion

To construct a detailed mathematical model that accounts for all redox states formed during electron and proton transport in complexes III and I, we used our rule-based methodology for automated construction of large systems of ODE [23]. This model further extends our methodology previously used to model the distribution of ^13^C isotopes in central metabolism [20]–[22], development of which occasionally coincided in time with that of similar rule-based methodology for signal transduction pathways [32], [33]. For the study of mitochondrial processes our methodology gives a deep insight into the mechanics of respiration and ROS production. Here, rule-based algorithms for mathematical description of mitochondrial respiration coupled to proton translocation and ΔΨ formation was linked to a classical kinetic model that accounts reactions of the TCA cycle, which provides succinate and NADH as substrates for respiration and substrate transport in mitochondria.

After fixing the ratios of forward and reverse rate constants for electron transport reactions, free parameters were defined by fitting of forward and reverse electron flows measured under various conditions. High variability of parameters with a good fit to experimental data precluded definition of their values. However, the levels of free radicals calculated in the model showed much less variability. Different sets of parameters with a good fit to experimental data define very similar patterns for free radicals formed in complexes I and III. Thus, the analysis gives a valid insight into the mechanism of respiration and ROS production, even without precise evaluation of the model parameters.

A substantial body of experimental data on mitochondrial ROS production cannot be satisfactorily explained within the current experimentally based paradigm. Some of these results were obscure, such as acceleration of succinate-driven ROS production after initial inhibition by rotenone (Figure 3). Others, such as a lower membrane potential in mitochondria fuelled by higher succinate concentration (Figure 9), were even counterintuitive. Calculation for mitochondrial constituents not measurable by current techniques represents a powerful tool for mechanistic explanation of accumulated data and for directing experimental research to test model predictions.

A body of evidence indicate that either FMNH [29], [30], or SQ bound to Qn sites of complex I [30], or reduced N2 centers [30], [34], [35] may be a major contributor to ROS production, depending on the tissue, substrate, energy demand and oxygen tension [36], [37]. The simulations revealed correlations between measured ROS production rates and levels calculated for each type of free radical. In this first step of the study we did not assume any explicit link between any specific radical and ROS, but qualitatively compared all of them, taken separately, with measured ROS production. However, the method, which we use, opens a direction for future studies of quantitative contribution of various radicals of electron transporters, and even specific species of complex I and III, into total ROS production.

The similarity between changes in the ROS production rate and in the levels of specific free radicals validates the model and also provides an insight into the mechanism of ROS production. Rotenone inhibition of ROS production in succinate-fueled mitochondria correlated with the free radicals formed in complex I, but not in complex III. Evidently, under the given conditions, reverse electron transport must contribute to free radical formation in complex I, although the net flux reducing NAD^+^ through complex I exists for only a very limited period of time.

In accordance with our previous study that revealed bistability for complex III [23], the extended model confirms the existence of two steady states for the same set of parameters. In one of these states (oxidized), mitochondria can develop a maximal rate of respiration, ΔΨ, and a capacity for ATP synthesis. This is the usual working state. In the presence of pyruvate high succinate concentrations can induce a switch of respiration to the reduced steady state, where lack of electron acceptors strongly restricts electron flow. The levels of free radicals in complex III greatly increase in this state, but decrease in complex I, in contrast. The switch to a more reduced state results in ΔΨ decrease. Indeed, we observed a ΔΨ decrease in isolated mitochondria in conjunction with an increase of succinate concentrations in the presence of pyruvate.

Q-cycle mechanism of complex III operation assumes bifurcation of electron flow at Qo site: one electron goes to Rieske center and further to complex IV, and another one reduces cytochrome b. This bifurcation of electron flow underlies the bifurcation between the two steady states. If in some moment the rate of first electron transition to Rieske center is higher than that for the second electron (because cytochrome b is reduced), semiquinones at Qo accumulate, thus preventing Qo liberation, binding and oxidation new molecules of ubiquinol, and thus limiting electron flow. In the case, shown in Figure 9A, greater proton leak resulted in greater transient discrepancy between the two electron flows at the point of bifurcation, which ultimately leaded to more significant inhibition of respiration and deeper descent of ΔΨ.

The decrease of ΔΨ, in the case shown in Figure 9B, is relatively small, however, in living cardiomyocytes a much higher decrease of ΔΨ can be observed, accompanied by high ROS production, and associated with mitochondrial permeability transition (MPT) [38]. Although the presented study does not touch a possible link between bistability and MPT, it puts forward some hypotheses, which verification in future can essentially clarify the mechanism of MPT. There are at least two phenomena, which do not find appropriate explanation in terms of current state of knowledge. First, the increase of permeability in this process does not increase electron flux and proton recirculation, as in case of uncouplers. Second, ROS production is high despite the decrease of ΔΨ. If we assume that the switch into the reduced state precedes MPT, both phenomena would find a natural explanation. This hypothesis, although not proved yet, opens avenues for deeper investigation of the MPT mechanisms.

The presented new insight into mitochondrial respiration was possible due to the application of novel methodology of modeling that allowed a detailed mathematical description of mitochondrial respiration. The phenomenon of bistability, predicted based on this methodology, has a potential to be a basis of new paradigm for the mechanism of ROS production, which will initiate new research with outcome on academic and practical levels.

## Methods

The file executable in Linux, which runs the simulations, and the C++ code of the program could be downloaded free from http://www.bq.ub.es/bioqint/ros_model/plcb2010.cpp.tar.gz.

### Electron transport in complex III reflected in the model

The model of complex III described elsewhere [23] was used as a part of the extended model presented here. For each reaction two values, forward (K_f_) and reverse (K_r_) rate constants, were used as parameters. The order of magnitude of K_f_ was set based on [19] and then K_r_ was determined as described in [23] using midpoint electrochemical potentials, which determinations was variable and allowed refinement by fitting the data presented in “Results”. Table 2 summarizes the reactions and values of parameters for complex III that simulate the data.

**Table 2:** Reactions and rate constants for complex III.

**Electron transport**					
**1,Qo: Fe^3+^+QH_2_↔Fe^2+^+Q^−^+2H^+^**					
k_qp_FS_	200000	ΔE	50	k_rqp_FS_	28000
**2,c1: Fe^2+^+c_1_^ox^↔Fe^3+^+c_1_^red^**					
k_FS_c1_	528000	ΔE	33	k_rFS_c1_	143000
**3,Qo: Q^−^+b_L_^ox^↔b_L_^red^+Q**					
k_qp_bl_	90000	ΔE	80	k_rqp_bl_	4000
**4,b: b_L_^red^+b_H_^ox^→b_H_^red^+b_L_^ox^**					
k_bl_bh_	80000	ΔE	119	k_rbl_bh_	900
**5,Qi: Q+b_H_^red^→Q^−^+b_H_^ox^**					
K_bh-qn1_	100000	ΔE	29	K_rbh-qn1_	33000
**6,Qi: Q^−^+b_H_^red^+2H^+^↔QH_2_+b_H_^ox^**					
k_bh_qn2_	250000	ΔE	50	k_rbh_qn2_	25000
**7,c1: c_1_^red^→c_1_^ox^**					
k_c1c_	260				
**Binding – dissociation**					
**8,Qo: Qo+QH_2_↔QH_2_@Qo**					
k_qHbnd_	3700			k_rqHbnd_	2600
**9,Qi: Qi+Q↔Q@Qi**					
k_qnbnd_	7000			k_rqnbnd_	200
**10,Qo: Q@Qo↔Qo+Q**					
k_qpdis_	3600			k_rqpdis_	1000
**11,Qi: QH2@Qi↔Qi+QH_2_**					
k_qhnds_	4000			k_rqhnds_	2500
**12,Qo: O2+Q-@Qo→O2−+Q@Qo**					
k_ros_	0.02652				

Reverse rate constants marked (K_r_) are calculated from the respective forward rate constants and midpoint potentials (ΔE) as described in [23]. Although the reactions of electron transport are shown in simplified form as bimolecular, in fact they are performed (and simulated in the model) as transitions between the states of the whole complex (monomolecular). The units of rate constants are described in the legend of Table 1 ( s^−1^ for monomolecular reactions and s^−1^·(nmol/mg prot)^−1^ for the rate constants of bimolecular binding).

### Electron transport in complex I reflected in the model

The overall process catalyzed by complex I is oxidation of NADH coupled with ubiquinone reduction and pumping 4 protons from negative to positive side of the membrane:
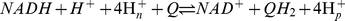
(I.0)This is a complex process that involves electron transport through a chain of intermediates coupled with proton translocations through inner mitochondrial membrane. The structure and mechanism of catalysis of complex I is reviewed in [39] and the data from this review are used for the construction of model of complex I.

It is assumed that proton translocation is a result of Q reduction (with proton binding) at the negative side and its oxidation (and proton release) at the positive side. If several protons are translocated per one electron, then this electron must pass several cycles of Q reduction and oxidation. Such mechanism, similar to that accepted for complex III, called Q-cycle, was suggested for complex I (see e.g. [17]). We constructed a model based on electron cycling that is in accordance with the measured stoichiometry of proton translocations per one electron passed through the chain.

The initial step of such transport is the oxidation of NADH coupled with the reduction of FMN; further, electrons from FMN pass through a relay of eight different iron-sulfur (Fe-S) containing centers [40], which possibly form a relay for electron transport from FMN to the last Fe-S center N2 (see e.g. the review [40], [41]). The Fe-S centers have similar midpoint potential close to that for FMN (E∼−350 mV) with an exception of N2, which is much more positive (−150 mV, [40]). In this model the relay of Fe-S centers is simplified, so that electrons pass from FMN directly to the N2 center, which can interact with quinones. In this way, two-electron transporter FMN and one-electron transporter N2 form the core of complex I, N2-FMN- FMN (referred as core).

The mechanism of interaction of N2 center with ubiquinone that results in the translocation of four protons from matrix to cytosol and one ubiquinol synthesized is not fully understood. Here we implemented in the model a proposed mechanism, which we consider as a working hypothesis that could be checked by the analysis of model behavior. In this way the model could serve as a tool for checking different possible mechanisms.

According to the EPR data [42], [43] there are two ubiquinone-binding sites; bound ubiquinones possess different EPR characteristics, one of them is fast- and another is slow-relaxing. The former one bound in oxidized form in the proximity of N2, in Qn site, could be reduced by N2 and bind protons taking them from negative (matrix side of the membrane) (indicated as Qn below). The other one, bound in the reduced form in Qp site, situated in the proximity of Qn, can interact with Qn-bound semiquinone releasing protons to the positive (cytosolic) side of the membrane. This interaction of two quinones in fact is in agreement with the idea outlined in [39] that complex I contains a single, but very large, binding domain for its hydrophobic substrate. Binding Qn and Qp gives additional three species of the complex I, Qn-Qn-N2-FMN- FMN, Qp-Qp-N2-FMN- FMN, and Qp-Qp-Qn-Qn-N2-FMN- FMN.

**Figure 10 pcbi-1001115-g010:**
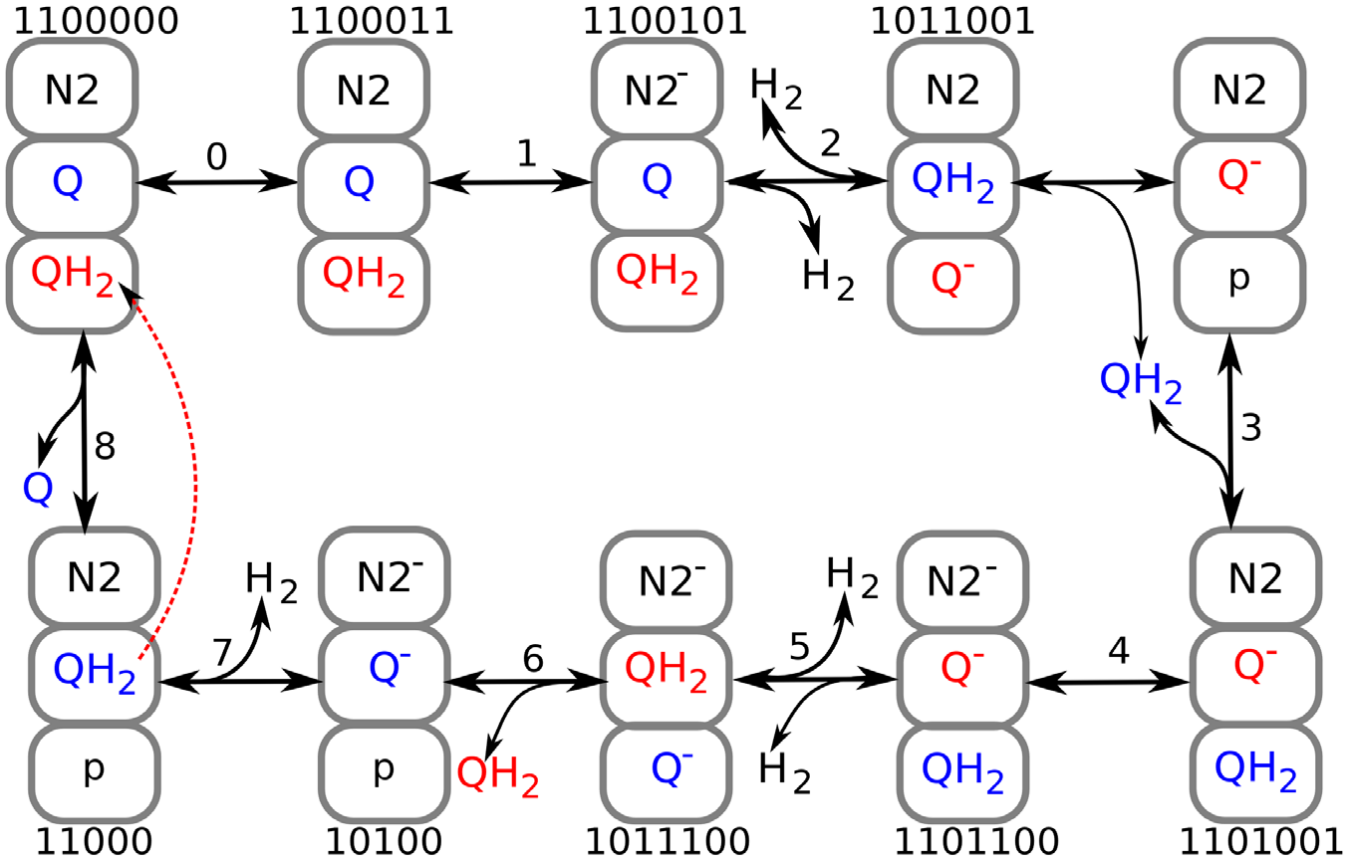
Interactions between N2 centers and quinones in complex I. Numbers above or below a species indicate the redox state of the complex as a combination of electron transporters. The last two digits indicate the presence (1) or absence (0) of two valence electrons of FMN (not shown graphically). The third digit from the right denotes the state of the N2 center, the next two digits from the right indicate the presence or absence of two valence electrons of Q/Q^−^/QH2 at the n-site. The next two digits from the right indicate the valence electrons of Q/Q^−^/QH2 at the p-site. Numbers 0–8 above arrows denote individual reactions. 0, FMN reduction by NADH; 1, electron transition from FMN to the N2 center; 2, electron transition from reduced N2 to n-site ubiquinone. This interaction results in electron transfer from p-side ubiquinol to n-side semiquinone, which is coupled to binding of two protons taken from the matrix side and release of two protons to the intermembrane space. 3, ubiquinol thus produced is released and p-site semiquinone changes its position, releasing the p-site, which binds the released ubiquinol; 4, n-site semiquinone takes an electron from p-site ubiquinol and forms ubiquinol, taking two protons from the matrix, while the p-site semiquinone formed releases two protons to the p-side of the membrane. 6, ubiquinol formed at the n-site dissociates and semiquinone bound at the p-site changes its location, binding to the n-site. 7, the non-paired electron of N2 is captured by n-site semiquinone, which subsequently takes two protons from the matrix and is converted to ubiquinol. 8, release of n-site bound ubiquinol, and binding of ubiquinol at the p-site and ubiquinone at the n-site.

The proposed mechanism of N2-ubiquinone interactions, which we implemented in the model, is shown in Figure 10, and the individual reaction steps are described in the legend. It satisfies the known stoichiometry of proton translocation and ubiquinone reduction (four protons translocated and one ubiquinol synthesized per two electrons taken from NADH).

### The elementary reactions of complex I simulated in the model

0. Reduction of oxidized FMN by NADH.

In traditional form this equation is expressed as
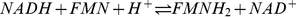
(I.0.0)Any of the forms of complex I with reduced FMN can receive two electrons from NADH, however, subsequent transitions require the interaction of three centers, N2, Qn and Qp. Therefore effective outcome produces only the reduction of FMN in the specie qnpc with ubiquinone bound at Qn and ubiquinol bound at Qp, which is reflected by binary number 1100000 corresponding to decimal 96. The reduction of FMN results in the production of redox state 1100011 (decimal 99):
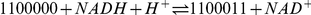
The forward and reverse reaction rates for this transformation are expressed in accordance with mass action law:
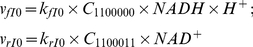
(I.0.1)Here, as described for the complex III, “0” designates oxidized and “1” reduced states.

The ratio of rate constants from I.0.1 could be found from the known redox potentials. Equilibrium constant for this reaction as a function of midpoint electrochemical potentials could be found from the condition of equality of electrochemical potentials at equilibrium:
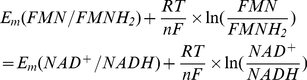
(I.0.2)

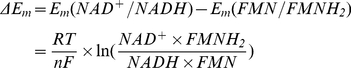
(I.0.3)since 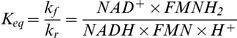, expression (I.0.3) could be rewritten as
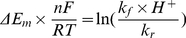
(I.0.4)

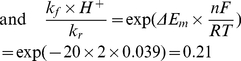
(I.0.5)taking into account that the difference between midpoint potentials for NADH (−320 mV) and FMN (−340 mV) [40] is ΔE_m_ = −20 mV.

1. Reduction of the N2 center by FMN (step 1 in Figure 7):
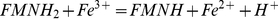
(I.1)First electron of FMNH2, which by convention occupied second position from the right in binary representation, passes to N2 converting 0 into 1 in the third position from the right:
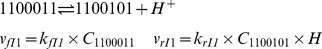
(I.1.1)The relationship between forward and reverse rate constants could be defined similar to (I.7). For the first transition at equilibrium
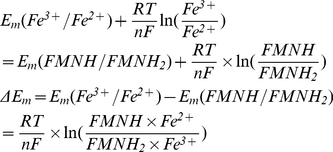
(I.1.2)since 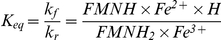, eq (I.1.2) can be written as
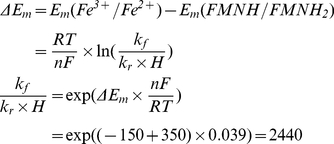
(I.1.3)since at pH = 7 Em(FMN^−^/FMNH_2_) = −350 mV [43], and Em for N2 iron-sulfur center Em = −150 mV [40].

2. Reduction of Qn by the reduced N2 center (first electron) and by QH_2_ bound at Qp center (second electron):
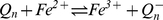
(I.2a)In binary form:
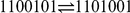
The semiquinone Q^−^_n_ is very active [17], so it reacts with QH_2_ bound at p-site:
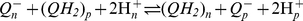
(I.2b)In binary form:
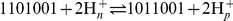
This reaction is symmetrical: p-side quinol and n-side semiquinone give p-side semiquinone and n-side quinol. The distance between the two quinone binding sites can be estimated as follows. Fast-relaxing semiquinone (bound to n-side oriented proton well) situated at the distance of ∼12 Å from N2, slow-relaxing semiquinone (bound to p-side oriented proton well) situated at the distance of ∼30 Å from N2 [42]. The distance between the bound quinones could be around 18 Å, which makes possible the interaction between them, taking into account the high energy of electron coming from FMN to Qn-bound quinone through N2 center. The assumption of such interaction fulfills the known stoichiometry of translocation of four protons and overall reduction of one ubiquinone coupled with the transport of two electrons through complex I.

We grouped together these two reactions:
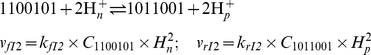
(I.2)Overall in this reaction the oxidation of N2 center is coupled with the reduction of Q_n_.
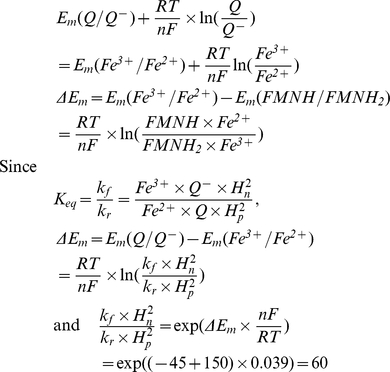
Here is considered that Em for N2 center is −150 mV [40] and Em for ubiqunone one-electron reduction is −45 mV [42].

3. Dissociation of QH_2n_ at n-site, transition of p-site SQ_p_ to the n-site and binding of dissociated QH_2n_ at p-site.

In this step the three reactions are combined: dissociation of QH_2_ formed at n-site, change of position of p-site bound semiquinone, and binding QH_2_ at p-site. Overall in binary form:
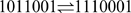
(I.3)and the forward and reverse rates are:
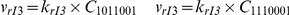



4. Second electron (from radical FMN^−^, which by convention occupied the right position) passes to N2 converting 0 into 1 in the third position from the right:
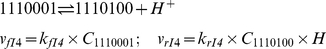
(I.4)


The transition of second electron characterized by the same ΔE_m_ as accepted in (I.1), but it is not related with proton binding or release, therefore the right hand side value of (I.1.3) equals to the ratio k_f_/k_r_.

5. Reduction of N2 by FMN in step 4 induces the interaction of n-site semiquinone with p-site quinol resulted in the production of n-site quinol and p-site semiquinone coupled with the translocation of two protons:
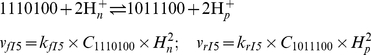
(I.5)The dissociation of n-site quinol produced and the change of position of p-site semiquinone:
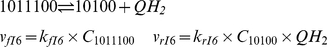
(I.6)


6. The reduction of n-site semiquinone by N2 coupled with the binding of two protons:
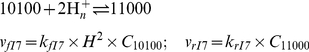
(I.7)In equilibrium
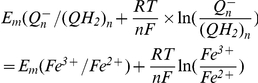


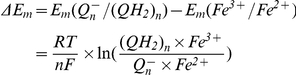
Since 
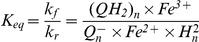
,
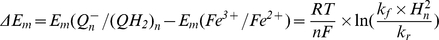
and the ratio of forward and reverse rate constants could be expressed as
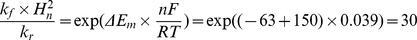
taking into account that Em for ubiquinol reduction in −63 mV [42] and Em for reduced N2 center oxidation is −150 mV [40].

7. QH2 dissociates, Q binds at n-site and QH_2_ binds at p-site, overall:
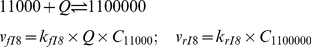
(I.8)Above, the ratios of forward and reverse rate constants for the redox reactions of complex I are defined and summarized in Table 3. The particular values were defined from fitting the experimental data presented in “Results” using these ratios as restrictions. In some cases the fitting required different value of midpoint potential. This may indicate the differences in the operation of complex I in situ and under the specific conditions of midpoint potentials determination. Recognizing the importance of this subject we leave its studying for future because it deserves a separate consideration.

**Table 3:** Rate constants for the reactions performed by complex I.

**FMN: FMN+NADH+H^+^↔FMNH_2_+NAD^+^**					
k_fI0_	640000	ΔE	−20	k_rI0_	880000
**N2: FMNH_2_+Fe^3+^↔FMNH+Fe^2+^+H^+^**					
k_fI1_	157000	ΔE	93	k_rI1_	4200
**N2: Q(n)+Fe^2+^↔Fe^3+^+Q−(n)**					
k_fI2_	770000	ΔE	60	k_rI2_	80000
**Qp-Qn, 1: Q^−^(n)+2H^+^(n)+QH_2_(p)↔**					
**QH_2_(n)+2H^+^(p)+Q^−^(p)**					
**2: QH_2_(n) Q^−^(p)↔QH_2_(p) Q^−^(n)**					
k_fI2_	23000			k_rI2_	150
**FMN: FMNH+Fe^3+^↔FMN+Fe^2+^+H^+^**					
k_fI4_	157000	ΔE	93	k_rI4_	4200
**N2: Q^−^n+Fe^2+^+2H^+^n = QH_2_+Fe^3^+**					
k_fI5_	190000	ΔE	107	k_rI5_	3000
**QH2: QH2@Qn↔Qn+QH_2_**					
k_fI6_	160000			k_rI6_	16000
**Q: Qn+Q↔Q@Qn**					
k_fI8_	200000			k_rI8_	3000

The units are the same as described in Table 1.

Although the mathematical description of complex I and complex III are similar, they differ in the strictness of rules for electron transport and proton translocation. For complex III the transition between two transporters allowed for any states of other transporters. This assumes participation of all 400 redox forms in electron transport. For complex I the rules accepted in the model allow participation in electron transport only several selected form. This illustrates the flexibility of methodology applied.

### Transmembrane electrochemical gradient of H^+^

Proton binding to ubiquinone at the matrix side of the membrane and their dissociation from ubiquinol to the intermembrane space results in the translocation of protons and arising the transmembrane gradient of H^+^ concentration and electric potential. As described above for complex I, the reactions (I.2), (I.5) and (I.7) reduce ubiquinone each time taking two protons from the matrix. In complex III the reduction of ubiquinone at Qi site by reduced cytochrome b_H_ is coupled with binding two protons taken from the matrix. The rate of this process (v_35_) is calculated as described in [23]. The electron flow (v_O_) through complex IV results in the reduction of oxygen with the uptake of two protons from the matrix and additional two protons are transferred from the matrix to cytosol. Proton leak (v_lk_) and ATP synthesis (v_syn_) return the protons transferred back to the matrix: v_lk_ is leak of protons through the membrane:
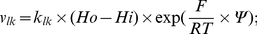
(H.1)v_syn_ is the reaction rate of ATP synthase, which uses the energy of three protons translocating them back to matrix to synthesize one ATP:
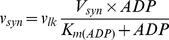
(H.2)The total rate of reversible uptake of the matrix protons is expressed as follows:
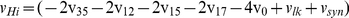
(H.3)The reactions (I.2) and (I.5) also release protons into the intermembrane space. For complex III the rate v_30_ release protons as described in [23]. The flux v_O_ transfers two electrons outside and leak and ATP synthesis consume the gradient:
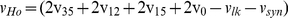
(H.4)The rates of proton translocations (H.1) and (H.2) change cytosolic (outside) and matrix (inside) proton concentrations (Ho and Hi) as described by the following differential equations:
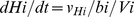
(H.5)

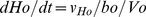
(H.6)Here bo, bi, Vo, Vi are the buffer capacity and volume of outer and inner intracellular space with regards to mitochondria respectively.

The differential equation for electric potential difference (ψ) used the same terms as that for proton concentration, but multiplied by a coefficient, which transforms the flux of ions into the change of electric potential:
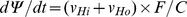
(H.7)where F is Faraday number (96000 cu/mol or 0.96·10−4 cu/nmol), C is electric capacity of the membrane (2·10−4 F/mg of protein, as computed based on [44].

### Connection of respiration with central energy metabolism

Substrates for respiration, i.e. NADH and succinate are produced in TCA cycle inside mitochondria and in the model the connection of this part of intracellular metabolism with respiration through these common metabolites is taken into account by the simulations of following reactions.

Since the emphasis of work described here is the operation of respiratory chain, the reactions of TCA cycle were simulated in simplified form, as linear function of each substrate. Such expressions assume that the substrate concentrations are far from saturation, which should be true for the most cases. In this case the usual hyperbolic dependence of enzymatic reactions on substrate concentrations is close to the linear dependence. On the other hand, this simplification allows to avoid such unfavorable situation, when choosing inappropriate Km makes reactions artificially insensitive to substrate changes. Therefore we used such assumption as a first approximation, which could be easily corrected with obtaining more information about the properties of system.

Pyruvate transport and transformation to acetil coenzyme A:
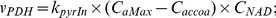
(T.1)Citrate Synthase :
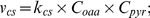
(T.2)here the conversion of pyruvate into acetyl coenzyme A, linked with NAD^+^→NADH transformation, is included in the same reaction.

The reactions converting citrate into succinate were joined together, taking into account that NAD^+^ is used in these reactions:
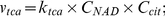
(T.3)Then succinate is transformed into fumarate in succinate dehydrogenase reaction, which reduces Q taking two protons from matrix:
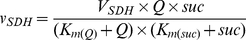
(T.4)Here the total content of reduced and oxidized ubiquinone is conserved at the levels defined by [45] (6 nmol/mg prot).

Succinate not only could be produced in TCA cycle but also transported from outside of mitochondria in exchange to fumarate or malate (which are lumped in one pool in the present version of the model):
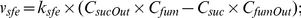
(T.5)Succinate could also be exchanged to phosphate:
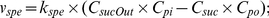
(T.6)Malate dehydrogenase reaction transforms lumped fumarate/malate pool into oxaloacetate producing NADH:
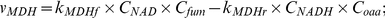
(T.7)Malic enzyme transforms malate into pyruvate producing NADH:
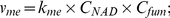
(T.8)The concentrations of metabolites were calculated by numerical solving the differential equations constructed using the above expressions for reaction rates:
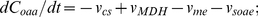
(T.9)

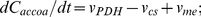
(T.10)

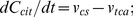
(T.11)

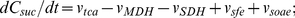
(T.12)

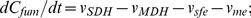
(T.13)The differential equation, which describes the evolution of NADH takes into account the stoichiometry of its production in TCA cycle and consumption by complex I (reaction (I.0) described above.
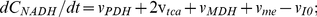
(T.14)The total concentration of NAD and NADH is assumed to be constant, so that NAD+, which defines the rates of TCA cycle reactions is computed as CNAD = CNADt−CNADH.

The reactions linked with electron transport and respective parameters are summarized in Table 4. In total, without the reactions of pyruvate transport and ATP synthase, which were switched out in accordance with experiments analyzed, this module contains 11 parameters.

**Table 4:** Reactions and rate constants for the reactions linked with respiratory chain.

**H^+^ leak:**		k_lk_	1500
**ATP synthase**			
V_syn_	0	K_mADP_	0.01
**pyr transport & pdh**			
k_pyrin_	630		
**accoa+pyr→cit**			
k_cs_	1300		
**2NAD^+^+cit→suc+2NADH+2H^+^**			
k_tca_	750		
**CII: suc→fum**		V_SDH_	170
Km_Q_	0.5	Km_suc_	0.1459
**Dicarboxylate exchange**			
k_sfe_	6.5		
**Excange suc↔Pi**			
k_spe_	270		
**NAD^+^+fum↔oaa+NADH+H^+^**			
k_MDHf_	460	*k_MDHr_*	460
**ME: fum+NAD^+^→pyr+NADH**			
k_me_	0.0003		

Maximal rates (Vmax) are expressed in (nmol/mg prot)/s, Km are expressed in nmol/mg prot, rate constants for bimolecular reactions are in s^−1^·(mg prot)^−1^, monomolecular reactions are in s^−1^.

As the presented equations show, although the expressions for reaction rates are simplified, the stoichiometry of succinate and NADH production and succinate transport is reflected precisely in the model and this was the most important for the presented step of study of the link between central metabolism and ROS production by electron transport chain and the role of reverse electron transport in this process.

### An algorithm for fitting experimental data

The whole model contains (22−6)+(18−7)+11 = 51 parameter (22 for complex III, 18 for complex 1, and 11 for the rest of reactions simulated). The six parameters of complex III and seven parameters of complex I are defined by the known values of midpoint potential. The other parameters were validated by fitting experimental data. To fit the experimental data our modification of Simulating Annealing algorithm was implemented in the way similar to that in [22]. The specificity of this algorithm was defined by the specificity of experimental data. The dynamics of NAD^+^ reduction was measured under the two different conditions, in the presence and absence of rotenone, an inhibitor of reduction/oxidation of quinones in complex I. The presence of rotenone was simulated by decreasing to zero the rate constants of step 5 in the reactions performed by complex 1 (k_f15_ = k_r15_ = 0), and the two conditions were fitted simultaneously for the same values of all other parameters. The procedure consisted of minimization of χ^2^, normalized sum of squares of deviations from experimental data. χ^2^ was calculated based on two simulations, first, normal conditions and, second, the presence of rotenone (k_f15_ = k_r15_ = 0, and all other parameters as in the first simulation).

The fitting algorithm made the following actions:

1. made the stochastic perturbation of given set of parameters (Vmax for the reactions of TCA cycle and substrate transport through the membrane)
2. performed coordinate descent, taking the parameters one by one and changing them in the direction, which decreased χ^2^
3. after reaching the local minimum of χ^2^ the program saved the respective set of parameters
4. returned back to step 1.

The cycles of perturbations and coordinate descent repeated thousands times and saved sets of parameters were analyzed: program read the saved sets with corresponding values of χ^2^, defined the best fit (absolute minimum of χ^2^), the set of parameters, corresponding to the best fit, and defined confidence intervals for the parameters using the criterion of Δχ^2^ [31].

### Experimental procedures

All procedures involving animals were approved by Children's Hospital of Pittsburgh and were in compliance with “Principles of Laboratory Animal Care” and the current laws of the United States.

**Brain mitochondria were isolated** from the cortex of adult Wistar rats. After removal, tissue was minced and homogenized in ice-cold isolation buffer I (IB I) which contained: 225 mM mannitol, 75 mM sucrose, 5 mM HEPES buffer (pH adjusted to 7.3 with KOH), 0.1 mg/ml fatty acid free BSA, 1 mM tetrapotassium EDTA and 12% Percoll. The homogenate thus obtained was carefully layered on the top of a discontinuous gradient of Percoll (24% and 42%) prepared using the same buffer. The preparation was then centrifuged at 16,000×*g* for 10 min. The fraction containing the mitochondria located between 42% and 24% Percoll was carefully withdrawn by a syringe and washed from Percoll twice by pelleting in IB I. The resulting mitochondrial suspension was diluted in isolation buffer II (IB II), which was similar to IB I, except for the concentration of EDTA (0.1 mM) and lack of albumin, and spun down at 12,000×*g* for 10 min. The deposit of mitochondria was homogenized in IB II at a final protein concentration of ∼20 mg/ml and stored on ice until use. The protein concentration in the mitochondrial samples was determined using a Protein Assay kit (Pierce Chemical Company, Rockford IL) according to the manufacture's instructions. Mitochondria prepared in this way were active for at least 5–6 hours, as determined by their ability to maintain a stable transmembrane potential in the presence of oxidizable substrates.

**Fluorescence measurements** were performed in a stirred cuvette mounted in a Shimatzu RF-5301 spectrofluorimeter maintained at 37°C. Mitochondria (0.2 mg/ml of protein) were added to 1.5 ml of the basic incubation medium that contained: 125 mM KCl; 2 mM KH_2_ PO _4_; 2 mM MgCl_2_; 10 mM Tris;10 mM HEPES (pH 7.0); 100 µM EGTA; and oxidizable substrates as indicated in a particular experiment. Concentration of rotenone, when indicated, was 1 mkM.

**Fluorescence of NAD(P)H** was measured at excitation/emission wavelengths 365 nm (slit 5 nm)/463 nm (slit 10 nm), respectively. To quantify the measurements a calibration curve was constructed using standard concentrations of commercial NADH.

**Hydrogen peroxide** was measured by fluorescence of Amplex red (2 µM), which increased upon oxidation to resorufin in the presence of 1 U/ml of horseradish peroxidase (HRP) as previously described [15]. Measurements were carried out at excitation/emission wavelengths of 560 nm (slit 1.5 nm)/590 nm (slit 3 nm), respectively. Amounts of H_2_O_2_ released by mitochondria were estimated by constructing calibration curves using known H_2_O_2_ concentrations in the standard incubation buffer together with Amplex red and HRP, but without mitochondria.

**Mitochondrial transmembrane potential**, ΔΨ_m_, was estimated using fluorescence quenching of the cationic dye safranine O. Since polarized mitochondria have a negative charge inside, positively charged molecules of safranine O are accumulated inside the matrix; increase in dye concentration inside the matrix leads to fluorescence quenching, thus, a decrease in fluorescence corresponds to an increase of membrane potential. The excitation wavelength was 495 nm (slit 3 nm) and emission 586 nm (slit 5 nm), and the dye concentration used was 2.5 µM [15].

**Mitochondrial respiration rates** were measured by an Oroboros High Resolution Respirometer (Innsbruck, Austria) in a stirred 2 mL chamber at 37°C in the same incubation media as indicated above. Oxygen sensor was calibrated at each experiment according to the manufacture's instructions. Calculations of respiratory rates were performed by software built into the instrument.

## Supporting Information

Figure S1 The scheme of reactions performed by complex III as it is generally accepted (considered in Selivanov et al, 2009). One of two electrons taken from ubiquinol (QH2), which releases its two protons into the intermembrane space, recycles through cytochromes bh and bl reducing another quinone. The other electron continues its way to oxygen through cytochromes c1 and c and complex IV. Complexes I and II provide QH2. The reactions 0–12 are described in detail in the text.
(0.04 MB TIF)

Figure S2 Simulation of time course of reduction of cytochromes bH (thick grey line) and c1 (thin black line), and ubiquinone (dashed line). This simulation was made using initial set of parameters. Ordinate represents the content of reduced forms in nmol/mg of protein, time units are arbitrary.
(0.02 MB TIF)

Figure S3 Simulation of time course of reduction of cytochromes bH performed with various values of dissociation constants for Q ans QH2 species. Curve 0 calculated with initial values of parameters taken in (Selivanov et al, 2009), curve 1 calculated with the tenfold decrease of Kd for QH2 and Q binding at Qo and Qi respectively, and the tenfold increase of Kd for Q and QH2 dissociation at Qo and Qi respectively. All the changes favor forward direction of Q-cycle. Curve 2 calculated favoring the reverse direction of Q-cycle by the tenfold decrease of initial value of Kd for QH2 dissociation, and all other parameters as for curve 1.
(0.03 MB TIF)

Figure S4 Simulation of time course of reduction of cytochromes bH performed with various combinations of ΔEm for the first an second electron transitions from bH to Q at Qi. In all presented cases Em( bHox/bHred) = 61 mV. Curve 0 is the same as in Figure 3, Curve 1 is calculated accepting Em(Q/Q−) = 45 mV and Em(Q−/QH2) = 150 mV (Rich, 1984). Curve 2 is calculated accepting Em(Q/Q−) = 90 mV and Em(Q−/QH2) = 16 mV (Covian, 2007). All other parameters are the same for all curves with the values given in (Selivanov et al, 2009).
(0.03 MB TIF)

Table S1 Sensitivity of simulation of mitochondrial respiration with regards to the parameters. First column gives the list of parameters, next four columns give the relative change of respectively dynamics of NAD+ reduction in the absence and presence of rotenone, uncoupled respiration fueled by succinate, and pyruvate/malate. Next four columns give the relative change of SQ at Qo site of complex III, in the same four simulations as above, then, relative change of SQ at Qn site of complex I, then FMNH, and finally, reduced N2 centers. The highest changes marked black.
(0.03 MB XLS)

Text S1 Analysis of triphasic dynamics of cytochrome bH reduction using the model of Complex III from our publication (PLoS Comput Biol 2009, 5(12): e1000619) for the validation of parameter.
(0.08 MB PDF)
