# Supplementary figures and images for: Reactive Oxygen Species Production by Forward and Reverse Electron Fluxes in the Mitochondrial Respiratory Chain
Source: PLoS Comput Biol. 2011 Mar 31;7(3):e1001115. doi: 10.1371/journal.pcbi.1001115 (PMC3068929; doi:10.1371/journal.pcbi.1001115)

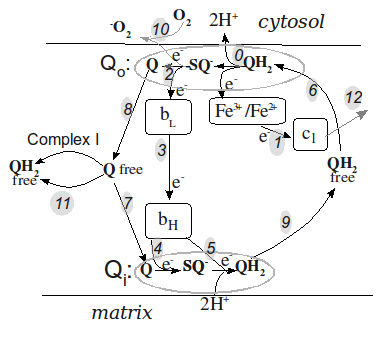

Supplement: Figure S1 — The scheme of reactions performed by complex III as it is generally accepted (considered in Selivanov et al, 2009). One of two electrons taken from ubiquinol (QH2), which releases its two protons into the intermembrane space, recycles through cytochromes bh and bl reducing another quinone. The other electron continues its way to oxygen through cytochromes c1 and c and complex IV. Complexes I and II provide QH2. The reactions 0–12 are described in detail in the text. (0.04 MB TIF) [file pcbi.1001115.s001.tif]

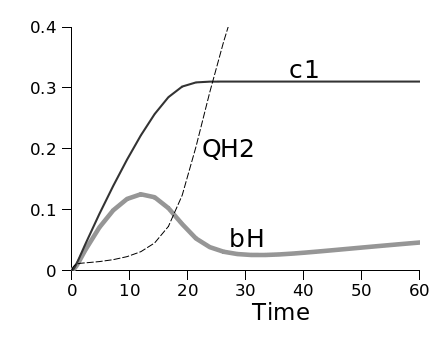

Supplement: Figure S2 — Simulation of time course of reduction of cytochromes bH (thick grey line) and c1 (thin black line), and ubiquinone (dashed line). This simulation was made using initial set of parameters. Ordinate represents the content of reduced forms in nmol/mg of protein, time units are arbitrary. (0.02 MB TIF) [file pcbi.1001115.s002.tif]

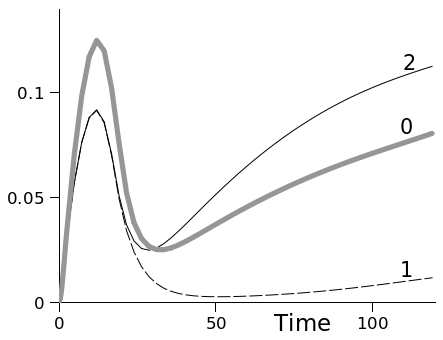

Supplement: Figure S3 — Simulation of time course of reduction of cytochromes bH performed with various values of dissociation constants for Q ans QH2 species. Curve 0 calculated with initial values of parameters taken in (Selivanov et al, 2009), curve 1 calculated with the tenfold decrease of Kd for QH2 and Q binding at Qo and Qi respectively, and the tenfold increase of Kd for Q and QH2 dissociation at Qo and Qi respectively. All the changes favor forward direction of Q-cycle. Curve 2 calculated favoring the reverse direction of Q-cycle by the tenfold decrease of initial value of Kd for QH2 dissociation, and all other parameters as for curve 1. (0.03 MB TIF) [file pcbi.1001115.s003.tif]

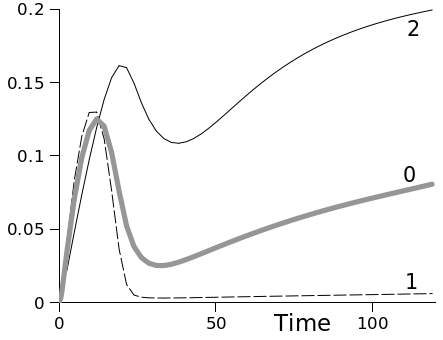

Supplement: Figure S4 — Simulation of time course of reduction of cytochromes bH performed with various combinations of ΔEm for the first an second electron transitions from bH to Q at Qi. In all presented cases Em( bHox/bHred) = 61 mV. Curve 0 is the same as in Figure 3, Curve 1 is calculated accepting Em(Q/Q−) = 45 mV and Em(Q−/QH2) = 150 mV (Rich, 1984). Curve 2 is calculated accepting Em(Q/Q−) = 90 mV and Em(Q−/QH2) = 16 mV (Covian, 2007). All other parameters are the same for all curves with the values given in (Selivanov et al, 2009). (0.03 MB TIF) [file pcbi.1001115.s004.tif]
