# Supplementary material for: Reactive Oxygen Species Production by Forward and Reverse Electron Fluxes in the Mitochondrial Respiratory Chain
Source: PLoS Comput Biol. 2011 Mar 31;7(3):e1001115. doi: 10.1371/journal.pcbi.1001115 (PMC3068929; doi:10.1371/journal.pcbi.1001115)
Supplement: Text S1 — Analysis of triphasic dynamics of cytochrome bH reduction using the model of Complex III from our publication (PLoS Comput Biol 2009, 5(12): e1000619) for the validation of parameter. (0.08 MB PDF) [file pcbi.1001115.s006.pdf]

Automated construction of large kinetic models: mitochondrial respiratory complex III

In the presented example the model of mitochondrial respiratory complex III, which was constructed based on the known biochemical mechanisms of electron and proton transport, simulated the experimentally measured triphasic reaction of cytochrome bH reduction by succinate in isolated succinate-cytochrome c reductase. These simulations validated the model and algorithms for its automated construction.

Multienzyme complexes, or multiple regulatory sites, which are of great importance in systems biology, require the construction of large number of differential equations. Even if such a complex has just several components, the combination of various states of each components result in huge number of states for the whole complex, and, respectively, equations to describe their evolution. An example of such multienzyme complex could be respiratory complexes in mitochondria.

The operation of respiratory complex III was simulated here using the basic principles of automated differential equations construction as described in (Selivanov et al, 2009) and schematically shown in Figure S1. The biochemical mechanism of electron transport and proton translocation for complex III, known as "Q-cycle" (Mitchell, 1975), was implemented in this model. The specific triphasic behavior of cytochrome bH reduction in the isolated complex is a direct consequence of Q-cycle mechanism (Tang and Trumpower, 1986). The origin of each phase of this reaction was analysed in details in a nice theoretical work (West, 1989). Here we simulated this reaction to show that the model reproduces this direct consequence of Q-cycle mechanism as a validation of our algorithms for model construction.

Simulation of particular experiments

In experiments, evidently, could not be measured all the forms, which are accounted by the model, although the whole distribution of redox form defines the experimental measurement. To compare the result of calculation with experimental data, the program summarizes the calculated concentrations of redox form, so that the summarized value corresponds to that measured. For instance if reduced cytochrome b_H is measured, the program summarizes all concentrations of all species for which b_H reduced, e.g. for core it summarizes all forms 1xxx, etc. The obtained value of reduced b_H could be compared with respective experimental data.

Implementation

In a one of fundamental experimental works that reveal the main properties of complex III operation, it was shown that the reaction of cytochrome b_H reduction by succinate in isolated succinate-cytochrome c reductase (complex III + complex II) has unusual triphasic behavior (Tang and Trumpower, 1986). In the original experimental study it was shown that such behavior is a direct consequence of Q-cycle

mechanism. Later, using fast equilibrium simulations, West (1989) had elucidated the mechanics of reduction, oxidation and subsequent ultitate reduction of b_H. Here we also simulated this triphasic reaction in order to check, whether our automatically constructed model correctly reflects the basic biochemical mechanism, which underlies such unusual behavior. Moreover, reproducing the experimentally observed behavior, we show a way of defining the proper parameters of system.

Model simulation of triphasic cytochrome b_H reduction by succinate in isolated succinate-cytochrome c reductase.

Figure S2 shows a model simulation of cytochromes b_H and c_1 and ubiquinone reduction by succinate in the isolated system of complex III and complex II (there is no acceptors for c_1), calculated with initial set of parameters. This initial set of parameters allows to reproduce the triphasic behavior of b_H reduction.

As it was proved experimentally (Tang and Trumpower, 1986) and theoretically (West, 1989), first phase of b_H reduction proceeds in accordance with the conventional way of net electron flow in complex III: ubiquinol produced by succinate dehydrogenase and bound at Qo site delivers its first electron to Fe^{3+} of Rieske center, which then reduces c_1. The second electron of ubiquinol passes to heme b_L and then reduces b_H. In this way, in accordance with Q-cycle mechanism, reduction of c_1 stoichiometrically linked with reduction of b_H.

In our simulation the initial rate of b_H reduction coincides with that for c_1. This indicates that the model correctly describes the separation of two electrons of QH_2 in accordance with Q-cycle mechanism. Later the reduction of b_H gets slower because reduced b_H again oxidized at Qi.

Since the experimental system lacks a respective electron acceptor, cytochrome c_1 ultimately becomes completely reduced. As Q-cycle mechanisms assumes, reduction of c_1 and thus block in electron flow coming from ubiquinol bound at Qo site in the same time blocks the delivery of second electron to cytochrome b. Thus, Qo site becomes blocked, which terminates the normal way of b_H reduction and first phase. Since b_H continues reducing ubiquinone at Qi site, the levels of b_H reduction decrease, which constitutes the second phase of cosidered triphase reaction.

During the first phase, ubiquinol, produced by succinate dehydrogenase, is oxidized at Qo site and therefore does not accumulate. After the Qo site gets blocked at the end of first phase, succinate dehydrogenase reaction results in the accumulation of ubiquinol. This accumulation induces third phase of b_H reduction, which proceeds through Qi site in the direction reverse to the normal net electron flow in Q-cycle.

The described sequence of events was revealed in the original paper (Tang and Trumpower, 1986). However, since such triphasic behavior strictly based of Q-cycle mechanism, the objective of presented

example was to show that our automated procedure correctly construct equations that correspond to the generally accepted mechanism of complex III operation. Moreover, in addition to the description of experimentally observed behavior, model simulation allowed revealing the details which were not measured, such as dynamics of ubiquinol production.

The analysis of characteristics of complex III operation

Figure S3 shows how the change of these values would affect the shape of trace of b_H reduction. If the dissociation constants change so that to favor the forward direction of Q-cycle, i.e. tenfold increase rate of binding of QH_2 at Qo and Q at Qi with respect to the rate of dissociation, and increase the rate of dissociation of Q at Qo and QH_2 at Qi with respect to the rate of binding, the third phase of b_H reduction slows down as curve 1 indicates (compared to curve 0, which is calculated for initial set of parameters, as in Figure S2). If with the set taken for curve 1 only Kd for QH_2 dissociation from Qi (reaction 9) decreases favoring the direction reverse to Q-cycle, the third phase of b_H reduction accelerates (curve 2). These simulations show that fitting a particular experimental curve could be very useful in the determination of Kd, which essentially affect the third phase of b_H reduction.

In the literature we found a large variability of ΔEm values for first and second electron transition from b_H to Q at Qi. This value varies even qualitatively, being negative (Rich, 1984) or positive (Covian et al, 2007) for the first electron, and positive (Rich, 1984) or negative (Covian et al, 2007) for the second electron. For the initial simulation we have taken ΔEm=0 for both cases, but variation of these values in simulations would help to define these parameters for a particular experiment.

Figure S4 shows the simulations of b_H reduction for the different combinations of ΔEm for the first an second electron transitions from b_H to Q at Qi.

If the values reported in (Rich, 1984) are taken for simulation, the phase 3 practically disappears. In contrary, if the values given in (Covian et al, 2007) are accepted, the third phase begins essentially before the completion of the second phase. It seems that the form of traces of b_H reduction is extremely sensitive to these parameters and fitting of a particular experiment would help to define them.

Thus, fitting particular experiments allows defining the parameters of the model and in this way to understand better the operation of respiratory chain in a specific experimental conditions.

References

Covian,R. et al. (2007) Asymmetric and redox-specific binding of quinone and quinol at center N of the dimeric yeast cytochrome bc1 complex. Consequences for semiquinone stabilization. J. Biol. Chem., 282, 24198-24208.

Mitchell P. The protonmotive Q cycle: a general formulation. FEBS Lett. 1975 Nov 15;59(2):137-9. PubMed PMID: 1227927.

Rich PR. Electron and proton transfers through quinones and cytochrome bc complexes. Biochim Biophys Acta. 1984 Apr 9;768(1):53-79. Review. PubMed PMID: 6322844.

Selivanov,V.A. et al. (2009) Bistability of mitochondrial respiration underlies paradoxical reactive oxygen species generation induced by anoxia. PLoS Comput Biol. 5(12):e1000619

Tang HL, Trumpower BL. Triphasic reduction of cytochrome b and the protonmotive Q cycle pathway of electron transfer in the cytochrome bc1 complex of the mitochondrial respiratory chain. J Biol Chem. 1986 May 15;261(14):6209-15. PubMed PMID: 3009448.

West IC. Triphasic reduction of bH and the absence of equilibration at the i-site of bc1 complex. Biochim Biophys Acta. 1989 Sep 28;976(2-3):182-9. PubMed PMID: 2551386.
